# Recognition and Regulation of T Cells by NK Cells

**DOI:** 10.3389/fimmu.2016.00251

**Published:** 2016-06-24

**Authors:** Katharina Pallmer, Annette Oxenius

**Affiliations:** ^1^Institute of Microbiology, ETH Zürich, Zürich, Switzerland

**Keywords:** T cell, natural killer cell, immune regulation, virus infection, autoimmune disease

## Abstract

Regulation of T cell responses by innate lymphoid cells (ILCs) is increasingly documented and studied. Direct or indirect crosstalk between ILCs and T cells early during and after T cell activation can affect their differentiation, polarization, and survival. Natural killer (NK) cells that belong to the ILC1 group were initially described for their function in recognizing and eliminating “altered self” and as source of early inflammatory cytokines, most notably type II interferon. Using signals conveyed by various germ-line encoded activating and inhibitory receptors, NK cells are geared to sense sudden cellular changes that can be caused by infection events, malignant transformation, or cellular stress responses. T cells, when activated by TCR engagement (signal 1), costimulation (signal 2), and cytokines (signal 3), commit to a number of cellular alterations, including entry into rapid cell cycling, metabolic changes, and acquisition of effector functions. These abrupt changes may alert NK cells, and T cells might thereby expose themselves as NK cell targets. Here, we review how activated T cells can be recognized and regulated by NK cells and what consequences such regulation bears for T cell immunity in the context of vaccination, infection, or autoimmunity. Conversely, we will discuss mechanisms by which activated T cells protect themselves against NK cell attack and outline the significance of this safeguard mechanism.

## NK Cells: Regulation, Effector Functions, and Education

Natural killer (NK) cells, presenting at a frequency of around 5% in the blood, belong to the family of group 1 innate lymphocytes (ILC1) and are functionally characterized by their cytotoxicity and their ability to produce cytokines, most prominently interferon γ (IFNγ). NK cells belong to the innate immune system, and they can react to rapid changes in host cells without prior sensitization. As part of the first line defense, they recognize and lyse virally infected cells and tumor cells. NK cells are activated *via* innate cytokines such as IL-2, IL-12, IL-15, IL-18, and type I IFNs as well as *via* the recognition of sudden cellular changes perceived *via* different inhibitory and activating receptors expressed on their surface (1–7). Additionally, direct triggering of toll-like receptors (TLRs) on NK cells can further stimulate their activation (8–10).

### Regulation of NK Cell Activity

Compared with T and B cells whose antigen receptors are highly variable and specific for a specific antigen, NK cells express various germ-line encoded activating and inhibitory receptors. Depending on the net balance of signals perceived by activating and inhibitory receptors, NK cells are either activated and exert effector functions or are restrained (11, 12).

Healthy cells constitutively express ligands for inhibitory receptors on NK cells in order to protect themselves against NK-mediated killing. Classical MHC-I molecules are expressed on every nucleated cell in the body and bind to the inhibitory receptor killer immunoglobulin-like receptors (KIRs) in humans and Ly49A C, D in mice, respectively. The non-classical MHC-I molecule HLA-E in humans and Qa-1 in mice binds to the heterodimeric inhibitory receptor CD94/NKG2A and CD48 binds to the inhibitory receptor 2B4, leading to a repressed state of the NK cell (13, 14).

Infected or malignant cells can downregulate MHC-I, also known as “missing-self hypothesis,” to become invisible for CD8 T cells; however, the loss of MHC-I ligands for inhibitory receptors on NK cells sensitizes these cells for NK-mediated killing. Conversely, overexpression of ligands engaging NK-activating receptors (“induced self-recognition”) also renders these cells NK cell targets (14, 15). Activating ligands are not expressed at steady-state, but tumorigenesis, virus infection, or DNA damage can activate stress pathways, leading to upregulation of various activating ligands that bind to NK cell-activating receptors and thereby promote NK cell activation, resulting in cytotoxicity and cytokine secretion (16). NKG2D is a well-studied NK cell-activating receptor, it has multiple cellular ligands including MHC-I homologs such as MHC class I chain-related proteins A and B (MICA and MICB) and UL16-binding proteins (ULBPs) (17). As a result of the activation of heat-shock transcription elements in the promoters of the genes, MICA and MICB are upregulated on NK target cells. The sensing of type I IFN can also trigger MICA and MICB expression on dendritic cells (DCs) (18, 19). Moreover, HCMV-infected cells upregulate MICA and ULBP3 (20, 21).

The DNAX accessory molecule-1 (DNAM-1 or CD226) is an adhesion molecule, which is expressed on multiple cells including NK cells. DNAM-1 serves as an activating receptor on NK cells, the engagement by its ligands poliovirus receptor (PVR), and nectin-2 leads to increased cytotoxicity in NK cells (22, 23). The cellular ligands of DNAM-1 are induced upon cellular stress (24, 25). Interestingly, regulatory T cells (Tregs) can also use DNAM-1–DNAM-1L interaction to modulate T cell responses, indicating that some receptors shared by innate and adaptive immunity are involved in regulating T cell responses (26).

Another family of NK cell-activating receptor is the natural cytotoxicity receptor family, consisting of NKp30, NKp44, and NKp46 in humans. Of note, NCR1 is the NKp46 ortholog and the only member of the NCR family in rodents (27). NKp30 and NKp46 are expressed on resting NK cells in contrast to NKp44 which is found only on activated NK cells. Cellular ligands for NKp44 are partly known and include proliferating cell nuclear antigen (PCNA) and mixed lineage leukemia 5 (MML5). Ligands which bind to NKp30 comprise HLA-B-associated transcript 3 (BAT 3) and B7-H6 (member of the B7 family of immunoreceptors) (28). However, the cellular ligands for NKp46 remain elusive (29, 30). Activating NK cell receptors may also directly be triggered by microbial constituents, such as Ly49H in C57BL/6 mice, recognizing the murine *Cytomegalovirus* (MCMV) encoded protein m157 and NCR1 recognizing influenza virus hemagglutinin proteins (31, 32) (Table 1).

**Table 1:** **Viral-derived ligands for NK cell receptors**.

Receptor	Ligand	Source	Effect of ligand/receptor interaction	Reference
NKp46	Viral HA and NA	Influenza virus, Poxvirus, Sendai virus, Newcastle disease virus	Activating	(31, 145–147)
NKp44	Viral HA and NA	Influenza virus, Sendai virus, Newcastle disease virus	Activating	(146, 148)
NKp30	Viral HA	Poxvirus	Inhibitory	(147)
	pp65	HCMV	Inhibitory	(149)
TLR7/8	Single-stranded RNA	HIV	Activating	(150)
Ly49H	m157	MCMV	Activating	(32)
KIR3DL1	NS-1	DENV	Activating	(151)

*HA, hemagglutinin; NA, neuraminidase; NS-1, non-structural protein 1; HCMV, human Cytomegalovirus; HIV, human immunodeficiency virus; MCMV, murine Cytomegalovirus; DENV, dengue virus*.

The fact that ligands for activating NK cell receptors are regulated *via* stress pathways and that microorganisms have evolved mechanisms to downregulate ligands for specific activating receptors might explain why there are different ligands for one receptor (33).

### Effector Functions

Key effector functions of NK cells comprise cytokine secretion and cytolytic granule-mediated cell apoptosis. The secretion of IFNγ and tumor necrosis factor (TNF) by NK cells promotes APC and phagocyte function, including enhanced phagocytosis, production of antimicrobial peptides, oxidative burst, and upregulation of MHC molecules. The granule exocytosis pathway is activated by the net balance of activation/inhibition signals conveyed by inhibitory and activating receptors and involves the secretion of granules which contain perforin and granzymes (16). Perforin/granzyme A and B trigger caspase-dependent and -independent death pathways. FasL and TRAIL are expressed on the surface of NK cells, which bind to the death receptors Fas (CD95) and TRAILR (DR4 and DR5), respectively. The engagement of FasL–Fas/TRAIL–TRAILR results in the induction of apoptosis of virally infected cells or tumor cells. Antibody-dependent cellular cytotoxicity (ADCC) can be exerted by NK cells when target cells are coated with IgG antibodies that bind the Fcγ receptor CD16 on NK cells, overriding inhibitory signals and triggering cytotoxicity and cytokine secretion (16, 34, 35).

### Education

As NK cells recognize mostly cellular ligands *via* their activating receptors, NK cells are potentially able to induce tissue damage. Thus, a tight regulation is required to avoid self-induced damage by NK cells. To this end, NK cells undergo a process called education, or “licensing,” describing a phase of NK maturation in which NK cells acquire effector functions and, at the same time, adapt their responsiveness to the steady-state expression levels of NK receptor ligands in host cells. For instance, mature NK cells recognize MHC-I molecules on the cell surface *via* their inhibitory receptors, resulting in a suppressed state of the NK cell toward healthy host cells (36). Surprisingly, the absence of MHC-I during NK cell education does not provoke overt NK cell activity, but NK cells appear to be hyporesponsive upon stimulation and fail to eliminate cells lacking MHC-I. This demonstrates that the environment, in which the NK education occurs, effectively determines the responsiveness of NK cells [(37, 38) and reviewed in Ref. (39)].

Interestingly, NK cells are not only killer cells as part of the first line defense but they also have the capacity to shape adaptive immunity by regulating T cell responses (40, 41). Of note, NK cells are not the only ILCs which are capable of modulating T cell responses. There is emerging evidence that other ILC subsets can contribute to shape T cell immunity, either by enhancing or suppressing the size of T cell immunity or by regulating the differentiation of T cell responses which was reviewed in Ref. (42, 43). In this review, we illustrate how specifically NK cells can regulate T cell immunity from a “T cell centric” point of view, and we will provide further insights into the relevance of NK–T cell interaction in various disease settings. In particular, we elucidate the role of NK–T cell interaction in acute and chronic viral infections. The profound understanding on how NK–T cell interaction occurs in different stages of T cell activation in different disease settings, and how it affects the size and quality of T cell responses might open new perspectives on the development of specific and powerful therapies.

## Stages in the Life of a T Cell

T cells need to perceive three signals for proper activation: antigen, costimulation, and cytokines. Antigen is processed and presented in the context of MHC class I and MHC class II molecules by APCs and determines the specificity of the response. Activated APCs provide further costimulation and cytokines, both needed for T cell activation. Among APCs, DCs that have been activated by engagement of pattern recognition receptors (PRRs) potently stimulate naive T cells (44). The signal of costimulation is provided by a number of molecules including CD28–CD80/86, CD27–CD70, OX40L–OX40, 4-1BB–4-1BBL, and RANK–RANKL (45–49). The specific cytokines required for T cell activation (i.e., proliferation, differentiation, and survival) result from the inflammatory milieu triggered by an infection or vaccination. In case of viral infections, most prominent proinflammatory cytokines are type I IFNs and IL-12, which have redundant functions as signal 3 cytokines (50). Integration of all three signals leads to fast T cell proliferation, known as clonal burst, reaching peak expansion before contraction and formation of a pool of memory cells.

At the peak of T cell expansion, T cells predominantly exhibit an effector phenotype (51, 52). Effector CD8 T cells can be split into two main subsets, a smaller subset of memory precursor effector cells (MPECs) that have the potential to become long-lived memory cells and short-lived effector cells (SLECs) that lack this ability (53).

### Impact of NK Cells on T Cells during Priming Phase

At steady-state conditions, NK cells are mainly excluded from the LN (54, 55). However, early during infections activated NK cells can enter into LN and localize in close proximity to T cells in the LN, enabling them to influence T cells during early stages of activation and thereby shaping the ensuing size and quality of the T cell responses. NK cells circulate in blood and migrate in a CXCR3-dependent, but CCR7-independent manner into activated LNs (56).

Since APCs are essential for cell activation, any changes in APC activation, maturation, and function are tightly associated with the emerging T cell response. Although NK cells were also shown to directly interact with T cells, this direct interaction seems to become more important after initial activation of the T cells. During the initial priming phase, T cell regulation by NK cells is mainly occurring in an indirect manner *via* modulation of APCs.

In the following section, we will elaborate in detail the different mechanisms of how DC–NK crosstalk influences the emerging T cell response.

#### NK Cells Promoting T Cell Immunity

Natural killer cells can be beneficial for mounting a T cell response by modulating DC function. NK cells act by different mechanisms depending on the DC subsets and the prevailing cytokine environment. In contrast to immature DCs (iDCs), which are found at steady state in peripheral tissues and in the blood, mature DCs (mDCs) migrate to the LN in a CCR7-dependent manner and have the ability to potently stimulate naive T cells. The conversion from iDCs to mDC occurs *via* sensing PAMPs *via* PRRs (57). mDCs are characterized by high level expression of costimulatory molecules, e.g., CD80, CD83, CD86, and MHC class II/human leukocyte antigen-DR (HLA-DR), as well by the secretion of proinflammatory cytokines such as IL-12 (58). To reveal interactions between DCs and NK cells at the early T cell priming phase, most studies with human cells used monocyte-derived DCs. iDCs are obtained by culturing monocytes in the presence of granulocyte macrophage colony-stimulating factor (GM-CSF) and IL-4. DC maturation is induced by the addition of LPS, IL-1, type I IFN, or TNF (59).

Human *in vitro* experiments revealed that DCs promote NK cell activity *via* the secretion of cytokines such as type I IFN, IL-12, or TNF. In return, NK/DC interaction leads to NKp30 engagement, resulting in TNF and IFNγ production by the NK cells, which further promotes DC maturation (58, 60, 61). Besides cytokines (IL-12, TNF, and IFNγ), mDCs can promote IFNγ production, CD69 expression, and cytotoxic functions of NK cells in a cell-to-cell contact-dependent manner. Ligands for the activating receptor NKG2D on NK cells, MICA and B, are expressed on human monocyte-derived DCs and contribute to CD69 expression on NK cells (58, 62, 63).

This bidirectional crosstalk can secondarily influence T cell differentiation. Specifically, the differentiation of naive CD4 T cells into IFNγ secreting T_H_1 T cells is promoted by NK cells, shown by transwell experiments *in vitro* using human myeloid blood DCs (64). Similarly, NK cells can also directly shape, possibly without direct DC crosstalk, the quality of T cell responses *via* their cytokine secretion. A murine *in vivo* study demonstrated that after activated NK cells migrated to the LN in a CXCR3-dependent manner where they served as an early source of IFNγ that was essential for Th1 polarization of naive CD4 T cells (56). In a mouse model of *Leishmania major* infection, the blockade of TGFβ signaling in NK cells led to increased IFNγ secretion by NK cells, promoting the differentiation of naive CD4 T cells into Th1 cells, leading to improved pathogen control (65).

Another mechanism how NK cells indirectly influence the emerging T cell response during the priming phase of T cells *via* enhancing cross-presentation. NK cells promote DC cross-presentation by killing target cells, leading to the release of antigens that can be taken up by DCs and presented *via* MHC-I to T cells, resulting in an increased CD8 T cell response. Specifically, transfer of allogenic B cells in mice resulted in NK-mediated killing that promoted endocytosis of apoptotic bodies by CD8^+^ DCs and MHC I presentation of respective antigens (66). Similarly, *in vivo* killing of OVA-expressing splenocytes by NK cells resulted in better priming of CD8 as well as CD4 T cells due to NK-mediated release of antigen. Here, NK-mediated cytotoxicity promoted CD4 T cell responses, which was crucial to support CD8 T cell responses and strong IgG responses (67). Also in human cells, NK cell-secreted IFNγ and TNF induce cross-presentation of tumor cell-derived antigens by monocyte-derived DCs, promoting the induction of a tumor-specific CD8 T cell response (68).

Several studies have reported that activated NK cells specifically kill iDCs, while sparing mDCs. Albeit the direct killing of iDC might have a negative impact on the emerging T cell response, it is more likely that the killing of iDCs might be beneficial for robust T cell immunity. This DC selection, known as DC editing, might serve as a quality control to guarantee the survival of DCs expressing sufficient costimulation molecules needed for successful T cell priming or to ensure that effective T cell priming is stopped after resolution of infection-induced inflammation (61, 69).

Human NK cells, activated with IL-2 *in vitro*, killed, and produced IFNγ when encountering iDCs. In contrast, lysis of mDCs occurred only when inhibitory ligands (HLA molecules) were blocked, showing that mDCs protect themselves against NK cell attack by KIR engagement. NKp30 engagement was mainly responsible for detection and elimination of iDCs. However, NKp30 was also shown to induce maturation and favor DC activation, and it is currently unclear how this differential role of NKp30 in NK cells is regulated (28, 69). The *in vitro* finding that human iDCs are preferably lysed by NK cells was confirmed in a tumor mouse model *in vivo*. Impaired tumor-specific T cell immunity was related to the missing deletion of iDCs in the absence of NK cells (70). NK cells can kill iDCs due to lacking expression of the inhibitory ligand Qa-1 in contrast to mDCs that upregulate its expression. Interestingly, iDCs were also killed under inflammatory conditions (71).

By contrary, it was also reported that iDCs can promote T cell immunity: in a DC-based vaccine model in mice prevention of TRAIL-mediated iDC killing by NK cells enhanced antigen-specific T cell responses (72). However, these studies relied on adoptive transfer of *in vitro* cultivated DCs (LPS treated, mature, monocyte-derived DCs and untreated, immature, DCs). To what extent these *in vitro* cultivated iDCs resemble *in vivo* iDCs remains to be demonstrated (69, 73).

**Figure 1:**
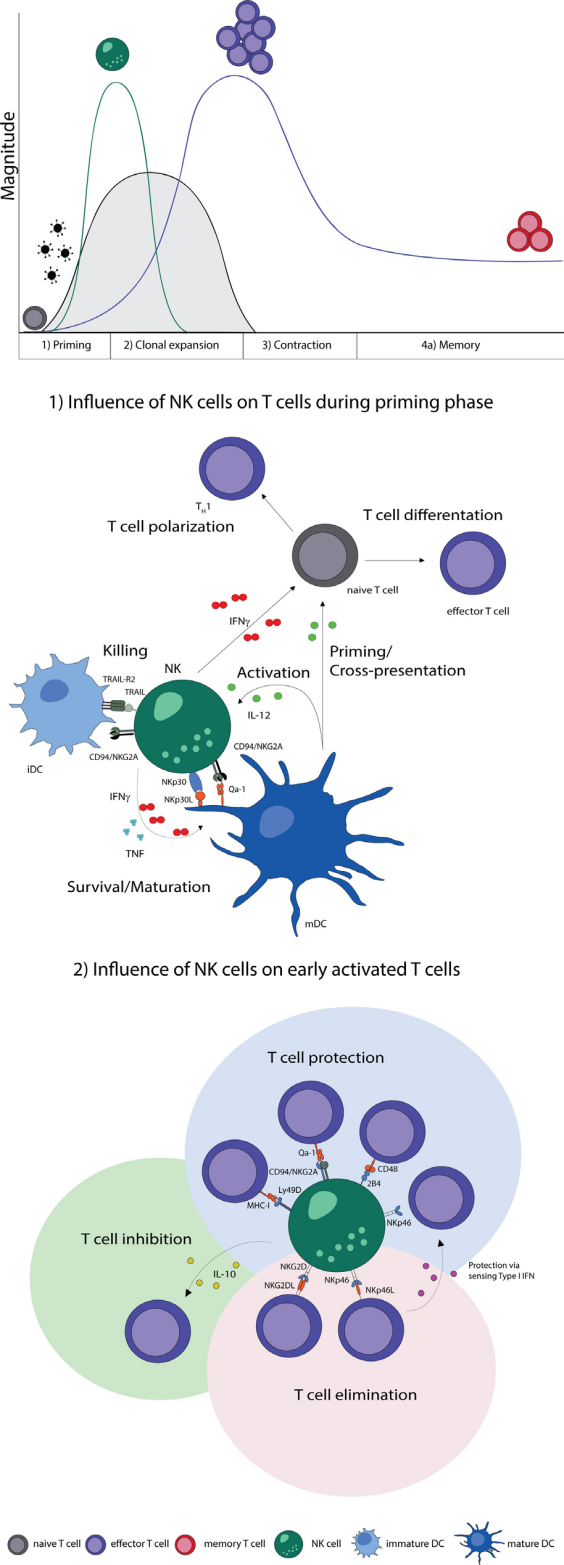
**NK–T cell interaction during early phases of viral infections**. Naive T cells (gray) are primed by DCs, expand, and differentiate into effector T cells (purple), contract and form memory T cells (red). Activation of NK cells and T cells show a temporal and microanatomical overlap early during infection allowing T cell regulation by NK cells. (1) Early during infection NK cells can directly regulate T cells *via* the secretion of cytokines. Indirectly, NK cells modulate DC numbers and function, which affect T cell responses. The bidirectional NK–DC crosstalk is achieved by cytokine secretion or *via* direct cell-to-cell contact, resulting in either positive or negative consequences for T cells. (2) Early activated T cells can be recognized and inhibited (green shading) or eliminated (red shading) by NK cells *via* cytokines or directly in a cell-to-cell-dependent manner. T cells shield themselves by upregulating ligands for inhibitory NK cell receptors (blue shading). Abbreviations: IFNγ, interferon γ; TNF, tumor necrosis factor; IL-12, interleukin 12; IL-10, interleukin 10; type I IFN, type I interferon; NKp30L and NKp46L, ligands for the NK cell-activating natural cytotoxicity receptors; NKp46 and NKp30, NK cell-activating natural cytotoxicity receptors; NKG2DL, ligands for activating NK cell receptor; NKG2D, activating receptor of NK cells; TRAIL-R2, tumor necrosis factor receptor; TRAIL, ligand for tumor necrosis factor receptor; CD94/NKG2A, Ly49D, and 2B4, inhibitory NK cell receptors; Qa-1, MHC-I, and CD48, inhibitory receptor ligands; T_H_1, T helper cell type 1; iDC, immature dendritic cell; mDC, mature dendritic cell.

In summary, NK cells improve maturation, effector functions, and cross-presentation of DCs and are thereby able to promote T cell responses. Moreover, NK cells can secrete cytokines during T cell priming, which promote the differentiation of naive CD4 T cells to Th1 cells. The elimination of iDCs might be a possible control mechanism to select immunogenic DC, which provide sufficient costimulation needed for the effective stimulation of naive T cells (Figure 1).

#### NK Cells Impairing T Cell Immunity

Negative impact of NK cell on T cells during priming can be either mediated by directly affecting T cells or indirectly *via* DC modulation.

Even though killing of iDCs might have a positive effect on T cell priming, direct killing of mature APCs, especially DCs, results in diminished antigen availability and thereby represents a mechanism how NK cells can negatively regulate T cell responses.

An *in vivo* mouse study revealed that in the absence of NK cells APCs show improved capacity to stimulate CD8, but not CD4 T cells early during LCMV infection and this enhanced virus-specific CD8 T cell response resulted in viral clearance of a usually persistent infection. The beneficial effects could only be observed when NK cells were depleted within first 2 days of infection and were not due to enhanced costimulation provided by APCs, but most likely due to increased numbers of APCs in NK cell-depleted mice (74). In the context of MCMV infection in C57BL/6 mice, the MCMV protein m157, that is directly recognized by L49H on NK cells, triggered NK-mediated killing of MCMV-infected DCs. The resulting lower numbers of antigen-presenting DCs in this setting translated into impaired T cell immunity and hence protracted lytic replication (75). Furthermore, in MCMV infection, proinflammatory cytokines, such as IL-12, IFNγ, and TNF, and NK cell-activating receptors, such as NKG2D and NCR1, were shown to promote a bidirectional NK/DC crosstalk that supported CD4 T cell priming. Interestingly, IL-10 negatively regulated this crosstalk, such that only in absence of IL-10, NK cells supported DCs to more effectively prime MCMV-specific CD4 T cells, resulting in enhanced virus control (76).

*In vitro*- and *in vivo*-activated murine NK cells can acquire MHC-II proteins from interacting DCs *via* membrane transfer in a process known as trogocytosis. However, MHC-II- and costimulatory molecule-expressing NK cells did not promote but rather inhibited proliferation of CD4 T cells in presence of DCs, suggesting that they competed with antigen-presenting DCs for engagement of CD4 T cells (77).

In contrast, human MHC-II-expressing NK cells that also express costimulatory molecules typical for DCs, such as CD80/CD86, are capable to induce CD4 T cell proliferation (78–81). Thus, the crosstalk between DCs and NK cells can either support or impair initial activation of T cells, raising the question which factors decide about the outcome of the interaction. First of all, depending on the nature of the cytokines NK cells are exposed to, they can either promote or inhibit the polarization of T cells. While IL-12 and IL-2 can promote the maturation of DCs that are capable of priming IFNγ secreting T_H_1 T cells, regulatory cytokines, such as IL-10, lead to a reduction of the NK–DC crosstalk and to a decreased priming of virus-specific T cells (76, 82).

Moreover, another decisive parameter is the NK:DC ratio. While in a situation of a low NK:DC ratio, NK cells preferentially promote DC activation and enhance secretion of cytokines by the DCs, a high NK:DC ratio favors NK-mediated killing of autologous DCs (61). Besides this ratio also the density of receptors on the NK cell surface may decide about their impact in shaping T cell responses. For example, NK cells that express a high density of NCR1 showed an increased cytotoxicity against NK sensitive tumor cell lines compared with NK cells expressing lower levels of NCR1. However, if the density of activating receptors on NK cells is also decisive for their ability to directly kill T cells or APCs remains to be investigated (83).

Taken together, NK cells have the potential to regulate T cell responses during their priming phase directly *via* proinflammatory cytokines, such as IFNγ, as well as indirectly by eliminating or promoting APCs numbers and function by various mechanisms (Figure 1). Future studies are essential to elucidate the factors which decide whether NK cells promote DC maturation, leading to an enhanced T cell response due to efficient priming of naive T cells or whether NK cells eliminate DCs which leads to reduced T cell immunity.

### Early Activation and Clonal Expansion

The following paragraph focuses on effector T cells; how NK cells influence Tregs and *vice versa* is summarized in Box 1. After encountering its cognate peptide presented by an APC in presence of costimulation and cytokines, a T cell rapidly undergoes clonal expansion. NK cells have the ability to recognize and directly kill early activated T cells and can thereby determine the quality and magnitude of T cell responses, which can influence the course of infection (84–90).

Box 1 **Treg–NK cell interaction.**

Regulatory T cells (Tregs) are characterized by the expression of the master transcription factor Foxp3 and are able to control effector T cells responses by inhibiting innate and adaptive immunity (91). Lately, several studies reported that Tregs influence NK cells. In *Scurfy* mice, that fail to develop Tregs, NK cells are hyperproliferative and show enhanced effector functions, hinting toward an interaction between Tregs and NK cells – or alternatively, that NK cells are activated as a consequence of the increased proinflammatory milieu presenting in absence of Tregs (92). Also, under homeostatic conditions, Treg depletion results in hyperreactivity of NK cells in terms of CD69 expression and cytolytic functions and IFNγ secretion (93). In line with increased activation in the absence of Tregs, NK cells show an increased missing self-responsiveness toward target cells and rapidly respond to weak stimulation. This hyperactivation depends on high levels of available IL-2 produced by CD4 T cells after Treg depletion. Thereby, low levels of IL-2, as in the presence of Tregs, can serve as a limiting factor restricting NK cell activity (94). This was confirmed in a genetic model of type I diabetes in mice in which Treg depletion led to infiltration and proliferation of NK cells in the pancreas, accompanied by the secretion of IFNγ that promoted disease progression. In this setting, the lack of IL-2 consumption by Tregs led to the elevated levels of IL-2, promoting NK cell activity, and was not due to higher numbers of CD4 T cells secreting IL-2 (95). Interestingly, upon MCMV infection, the restricting effect of Tregs on NK cells was abolished and virus titers between Treg-sufficient and Treg-depleted mice were comparable. Thus, the Treg-mediated suppression of NK cells might be overridden by availability of high IL-2 levels during viral infections (93).

However, in hepatitis C virus (HCV) infection, NK cells were suppressed by Tregs, which was shown in a human *in vitro* study. In HCV, NK cells are known to kill activated hepatic stellate cells (HSCs), which promote fibrosis. NK cell-mediated killing could be inhibited by Tregs in a cell–cell contact-dependent manner involving the cytotoxic T lymphocyte antigen 4 (CTLA-4). Interestingly, the preincubation of HSCs with Tregs resulted in reduced expression of activating ligands, such as MICA/B on HSCs, leading to suppression of NK cell effector functions (96). Not only CD4 Tregs interact with NK cells but also regulatory DCs (regDCs), which is a special DC subset inducing tolerance under certain physiological settings. The interaction between regDCs and NK cells induced an alternative activation state in NK cells, characterized by low levels of IFNγ secretion. This suppression was attributed to the secretion of IL-10 by DCs and, surprisingly, by the engagement of the activating receptor NKp46 on NK cells (97). This opposing effect of NKp46 might be due to the fact that naive NK cells were used in this DC coculture, while other studies used activated NK cells or cocultured NK cells in presence of virus-infected DCs (98, 99). Collectively, these studies show that NK cells can be controlled reciprocally by regulatory immune cells, in particular by Tregs. This notion might be relevant in the setting of diseases in which Tregs are increased, such as in chronic viral infections or cancer (100–103).

In mice, IL-2-activated NK cells specifically detect and kill activated CD4 and CD8 T cells in a perforin-dependent manner. Activated NK cells discriminate between activated and naive T cells *via* the activating receptor NKG2D (84). Similarly, human-activated T cells are susceptible to NK-mediated killing by upregulating ligands for NKG2D. Furthermore, human T cells also upregulate ligands for the activating receptor DNAM-1, such as PVR, upon stimulation with superantigen. Only T cells in the S and G2/M phases expressed PVR which is in line with the fact that NK cells preferentially kill proliferating T cells (104). These human *in vitro* studies showed that autologous T cells can be killed by NK cells *via* DNAM-1 and NKG2D in a granule exocytosis-mediated manner, which might serve as a mechanism to control T cell responses (85, 104).

*In vivo*, the absence of NK cells prevented establishment of a chronic LCMV infection in mice (86, 87). Mechanistically, some studies suggested that CD8 T cells can be directly killed by NK cells in a NKG2D- and perforin-dependent manner, while others reported that NK cells killed quite selectively activated CD4 T cells, which subsequently led to reduced CD8 T cell numbers and function. The NK cell-mediated killing of CD4 T cells required perforin; however, the exact mechanism how NK cells recognize activated CD4 T cells was not revealed (86, 87, 105). The differences in mode of action of NK cells during LCMV infection reported in these studies might be based on the use of different viral strains and infection doses.

Natural killer cells can regulate T cells also in a contact-independent manner *via* the secretion of IL-10, leading to the suppression of allergen and Ag-induced T cell proliferation (106, 107). In LCMV infection, IL-10 secreted by NK cells can restrict the magnitude of CD8 T cell responses during persistent viral infection and can thereby limit immunopathology (108).

As activated T cells are prone to be recognized and eliminated by NK cells, T cells have evolved mechanisms to protect themselves against NK-mediated cytolytic attack. One mechanism how T cells can protect themselves is *via* sensing type I IFN because virus-specific CD4 and CD8 T cells deficient for type-I interferon receptor *(Ifnar)* were effectively eliminated by NK cells in a perforin-dependent manner upon acute LCMV infection, demonstrating that sensing of type I IFN-induced specific downstream pathways in virus-specific T cells leading to protection from NK cell recognition and hence promoted survival and expansion (89, 90). Indeed, *Ifnar*-deficient activated T cells express ligands for the activating receptor NCR1 on their surface and are recognized *via* NCR1 on NK cells and are killed by the release of perforin. Virus-specific wild-type T cells, which were able to sense type I IFN, safeguarded themselves and resisted the NK cell attack due to absent expression of NCR1 ligands (90).

Interestingly, not only activated T cells need to evade NK cell-mediated killing but also NK cells themselves. Recently, it was reported that *Ifnar*-deficient NK cells were also killed by activated NK cells in a perforin-dependent manner. NK cell killing of *Ifnar*-deficient NK cells was not NCR1, but NKG2D dependent (109). The independent findings that type I IFN sensing is associated with absent expression of NCR1 or NKG2D ligands on the surface of different immune cells suggests that type I IFN downstream signaling pathways, including STAT 1 signaling, seem to provide conserved protective mechanisms of how activated immune cells avoid NK-mediated killing. In future studies, it will be important to gain a more detailed knowledge about the regulation of expression of ligands for activating or inhibitory NK cell receptors on activated immune cells.

Besides the upregulation of activating ligands on T cells, the absence of inhibitory ligands is also a mechanism how T cells can be rendered targets for NK cell-mediated lysis early during activation. On this line, *Ifnar*-sufficient virus-specific T cells showed higher expression levels of classical and non-classical MHC-I molecules, ligands for inhibitory NK cell receptors, compared with *Ifnar*-deficient cells (89).

In addition, NLCR5, a transcription modulator promoting MHC-I expression, was shown to protect T cells against NK cell attack, as *NLRC5*-deficient T cells became targets for NK cells under inflammatory conditions due to strongly reduced MHC-I expression (110). Furthermore, the absence of the inhibitory receptor 2B4 on NK cells was associated with a prolonged persistence of LCMV because virus-specific CD8 T cells were killed by NK cells. Even though CD8 T cells expressed high levels of MHC-I, they could not shield themselves from NK cell-mediated attack, showing that the protective features of MHC-I and CD48 are non-redundant (111, 112). Of note, 2B4 can act as a coactivating receptor on human NK cells, in particular, in combination with CD16 (113) or in synergy with NKG2D, NKp46, and DNAM-1, resulting in enhanced cytotoxicity of naive human NK cells (114, 115). However, if 2B4 as a coactivating receptor on NK cells impacts on T cell regulation remains to be demonstrated.

Natural killer cells were also shown to diminish the number of CD4 T_FH_ cells and thereby to affect B cell responses. During an acute LCMV infection, NK cells killed CD4 T_FH_ cells in a perforin-dependent manner, resulting in a weak germinal center (GC) response and reduced titers of virus-specific antibodies (116). NK depletion early during a chronic LCMV infection enhanced the levels of virus-specific antibodies, thereby contributing to control of the infection. Even though the underlying mechanisms were not revealed so far, elevated numbers of CD4 T_FH_ cells suggested that NK cells promote viral persistence by suppressing not only CD8 T immunity but also CD4 T_FH_ cells and consequently humoral immunity (117).

Taken together, NK cells have the potential to shape T cell responses early during activation, mostly by curtailing T cell responses and humoral immunity (Figure 1). Why would such regulation of T and B cell responses by NK cells exist? One situation in which this regulation has physiological relevance is chronic viral infections when NK cell-dependent killing of T cells and suppression of T cell functions may prevent T cell driven immunopathology. Furthermore, infection with intermediate LCMV doses normally leads to detrimental immunopathology due to a strong T cell response, which is not sufficiently inhibited by NK cells. Depletion of NK cells, in this model, leads to an early enhanced T cell response that mediates control of the infection (86).

Finally, NK cells may ensure that only “correctly” activated CD8 T cells, which have received all three activation signals, survive. “Incorrectly” activated T cells (i.e., those that might have only perceived one or two signals) might become targets for NK cells. This hypothesis originates from the observation that LCMV-specific T cells that have not sensed signal 3 (i.e., type I IFN) are exquisite targets for NK cells. Such incorrectly activated T cell might potentially be harmful for the host, for instance, in case of autoreactive T cells. This is, however, at the moment, pure speculation that needs to be addressed experimentally.

### Contraction and Memory Formation

The peak in the life of a T cell is around day 8 after antigen activation before the population of expanded T cells contracts massively due to apoptosis of antigen-specific effector T cells. The decline of T cell numbers is necessary to allow the organism to respond to new pathogens and to avoid immunopathology (118). NK cells do not seem to be involved in the contraction phase because virus-specific T cells decline in NK-depleted mice to the same extent as in NK-sufficient mice after acute LCMV infection (90, 105). After the contraction phase, few antigen-specific T cells differentiate into long-lived memory cells, which are dependent on IL-7 and IL-15 for homeostasis (119, 120).

Even though the decision about T cell differentiation occurs early during T cell activation and not during the contraction phase, the effects of NK cells on long-term memory formation will be discussed briefly. At the peak of T cell expansion, the population of antigen-specific T cell can be divided into SLECs and MPECs, with the latter fueling the pool of long-lived memory cells. In the absence or presence of NK cells, virus-specific T cells differentiated into MPECs and SLECs, indicating that NK cells do not affect the establishment of both effector subpopulations. Hence, establishment of T cell memory occurs both in presence and absence of NK cells during the priming phase in the context of an acute LCMV infection – albeit total numbers of memory cells might be slightly reduced in presence of NK cells due to slightly reduced peak expansion (88, 90, 116). In mice, the lack of Qa-1 on T cells, which binds to the inhibitory receptor CD94/NKG2A on NK cells, led to reduced survival of CD4 T cells. Furthermore, memory formation was drastically reduced, suggesting that Qa-1 plays a role in CD4 T cell survival and memory development (121). In an OVA vaccination model in mice, memory CD8 T cell responses were increased in the absence of NK cells, which was associated with a better control of tumor growth (105).

Natural killer–DC interactions not only occur during primary immune responses but also during recall responses, which was shown in a secondary infection model using different OVA-expressing pathogens. Upon secondary infection with *Listeria monocytogenes*, memory CD8 T cells were activated by IL-12 and CXCL9 secreting XCR1^+^ DCs, leading to improved reactivation of memory CD8 T cells. Upon recall, NK cells were rapidly activated and provided an early source of IFNγ, boosting XCR1^+^ DC functions, which resulted in a stronger recall CD8 T cell response (122).

Collectively, there are some indications that NK cells have the potential to shape the differentiation of memory T cells – although such impact may actually take place during the priming period – and decide thereby about the quantity of memory T cells.

### Disease

In the following part, we provide an overview about NK–T cell interactions in specific disease settings. In particular, we emphasize on how NK cells affect T cells during chronic viral infections and how chronic viral infections can modulate NK–T cell interaction. We also extend the discussion on the role of NK–T cell interaction in autoimmune disorders.

#### Role of NK Cells during Chronic Virus Infections

During chronic active viral infections, “classical” memory T cell formation is impaired. Instead, virus-specific CD8 T cells are maintained as a population of actively cycling cells that depends on recurrent activation by cognate antigen (123). Functionally, the virus-specific CD8 T cells are markedly impaired in their effector functions, predominantly in their ability to produce inflammatory cytokines such as IFNγ and TNF. This hypofunctional state is termed “exhaustion,” and its genesis is related to recurrent antigen exposure and the expression of a number of coinhibitory receptors that downregulate signal transduction from the TCR (123). Even though the depletion of NK cells early during a chronic LCMV infection leads to morbidity and mortality, due to enhanced antiviral T cell immunity, NK depletion 2 or 3 weeks after the peak of cytotoxic CD8 T cells is associated with improved antigen-specific CD8 T cell response and better virus control and led to only weak immunopathological symptoms (86, 124).

In human immunodeficiency virus-1 (HIV-1) infection, NK cells are activated and upregulate the activating receptor NKp44, while CD4 T cells express a cellular ligand for NKp44 (NKp44L). The expression of NKp44L is induced by the HIV-1 envelope gp41 protein and renders CD4 T cells highly susceptible to NK-mediated lysis. Interestingly, NKp44L expression was only observed in uninfected CD4 T cells. In infected CD4 T cells, the HIV-derived protein Nef inhibited the upregulation of NKp44L on the surface. Thus, the downregulation of NKp44-activating ligands on infected CD4 T cells might serve as an immune escape mechanism, while NKp44 ligand expression on non-infected CD4 T cells may contribute to the overall depletion of CD4 T cells over the course of infection (125). In addition, Nef and the late viral factor Vpu induced the downregulation of PVR, which is a ligand for the activating receptor DNAM-1 on NK cells. The blockade of DNAM-1 and NKG2D together reduced killing of HIV-1-infected cells by NK cells, indicating that both DNAM-1 and NKG2D are involved in the regulation of NK cell recognition of HIV-1-infected cells (126). Another study in HIV-1-infected patients revealed that plasmacytoid DCs secreted type I IFN which induced NK cells to kill HIV-infected CD4 T cells with the engagement of NKp46 and NKG2D. In this setting, NK-mediated lysis represents an antiviral immune response contributing to control the infection (127). Chronic hepatitis B virus (CHB) infection is also characterized by activated NK cells and a decrease of T cell functions or clonal deletion of virus-specific T cells. Studies using human samples derived from CHB patients revealed that virus-specific CD8 T cells in the liver are NK cell targets as they expressed higher levels of TRAIL-R2 which engages TRAIL on NK cells, leading to NK-mediated killing. Surprisingly, only HBV-specific T cells were affected by NK regulation *via* the TRAIL-R2/TRAIL pathway, but not other virus-specific T cells such as Epstein–Barr virus (EBV) or *Cytomegalovirus* (CMV)-specific T cells (128). NK cells and T cells derived from CHB patients are both functionally impaired. Nucleos(t)ide analog (NUC) therapy can improve T- and NK-cell responses and resulted in a reduction of an inflammatory phenotype of NK cells derived from CHB patients, characterized by reduced expression of TRAIL, CD38, and Ki-67. The change to a quiescent phenotype in NK cells was accompanied by the restoration of effector functions of HBV-specific T cells under NUC therapy. NK depletion and blockade of TRAIL and NKG2D further ameliorated the HBV-specific T cell functions (129). These studies clearly demonstrate that NK cells play a role in regulating HBV-specific T cells during chronic HBV infection. The regulation of HBV-specific T cells by NK cells might be under certain conditions beneficial for the host due to diminished immunopathological consequences. The complex interplay of NK cells and T cells in CHB was reviewed in Ref. (130).

EBV, which belongs to the *herpesviridae* family, causes a persistent latent infection and has a prevalence of over 90% in the population. While, in most individuals, the infection occurs without symptoms, in around 10% the infection results in infectious mononucleosis (IM), which is related to massive expansion of EBV-specific CD8 T cells (131). A study using humanized mice for EBV infection presented the hypothesis that NK cells could limit T cell expansion by directly killing T cells and thereby preventing the development of IM. It is therefore speculated that EBV seronegative individuals that have low numbers of NK cells are more prone to develop IM (132).

**Figure 2:**
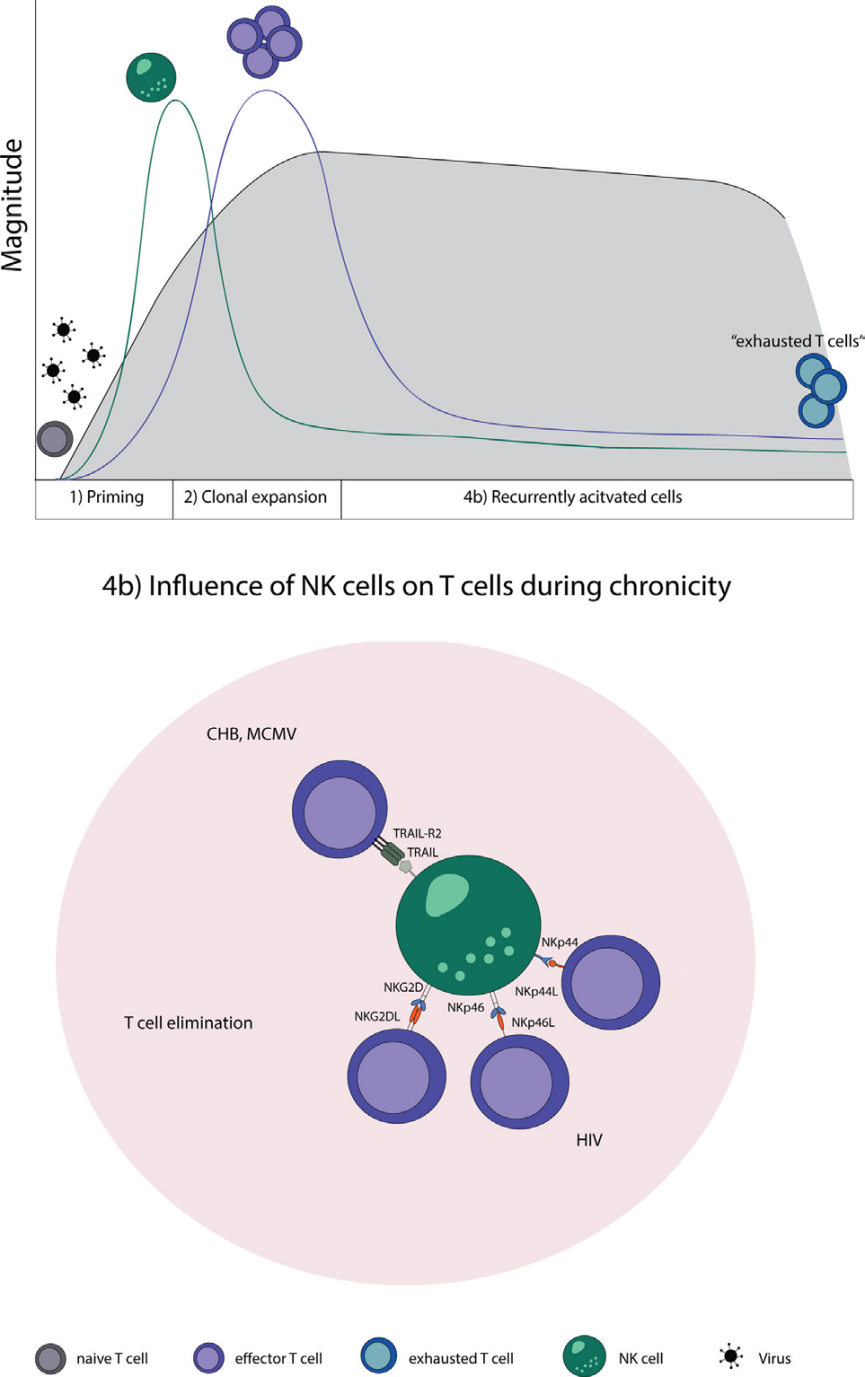
**NK–T cell interaction in chronic virus infections**. Naive T cells (gray) are primed by DCs, expand, and initially form effector T cells (purple) that transition during a chronic infection T cells to “exhausted T cells” (blue) due to constant restimulation by cognate antigen. (4b) NK cells recognize and eliminate T cells during a chronic state of viral infections by a direct cell-to-cell contact. Abbreviations: NKp44L and NKp46L, ligands for the NK cell-activating natural cytotoxicity receptors; NKp44 and NKp46, NK cell-activating natural cytotoxicity receptors; NKG2DL, ligands for activating NK cell receptor; NKG2D, activating receptor of NK cells; TRAIL-R2, tumor necrosis factor receptor; TRAIL, ligand for tumor necrosis factor receptor superfamily; CHB, chronic hepatitis B virus; MCMV, murine *Cytomegalovirus*; HIV, human immunodeficiency virus.

Natural killer cells are likewise involved in regulating T cell responses specific for MCMV, also a member of the *herpesviridae* family. During MCMV infection, NK cells kill activated CD4 T cells in the salivary gland. The elimination of activated CD4 T cells was dependent on TRAIL and led to a prolongation of the lytic virus replication; however, it prevented virus-induced autoimmunity at the same time (133). This shows that NK cells have, indeed, a regulatory role which has the ability to restrict strong adaptive immune responses in the setting of persistent viral infections (Figure 2).

#### Role of NK Cells in Autoimmune Disease

Natural killer cells are also involved in regulating autoreactive T cells. This was demonstrated in the mouse model for multiple sclerosis, experimental autoimmune encephalomyelitis (EAE). Depletion of NK cells was associated with a more severe form of EAE, characterized by the occurrence of relapses. The exacerbation of the disease was due to increased T cell proliferation and cytokine production (134). Besides the TRAIL–TRAILR pathway inducing apoptosis in autoreactive T cells, blockade of the Qa-1/CD94-NKG2A axis led to an amelioration of EAE due to the elimination of autoreactive CD4 T cells by NK cells in a perforin-dependent manner (121, 133).

The human CD56^bright^ NK cell subset is most prominent in peripheral lymphoid organs compared with the CD56^dim^ subset which mostly circulates. CD56^bright^ cells derived from healthy individuals could suppress autologous CD4 T cell proliferation under inflammatory conditions *via* the activation of NCRs and the secretion of granzyme. In comparison, CD56^bright^ derived from MS patients exhibited reduced granzyme secretion due to higher expression levels of the inhibitory ligand HLA-E on CD4 T cells and not due to altered NCR expression on NK cells (135). In addition, enrichment of CNS-resident NK cells in EAE had beneficial effects on the disease progression by suppressing T_H_17 cells (136). Paradoxically, the absence of IFNγ in a transgenic mouse model led to more severe symptoms in EAE, uveitis, and arthritis. In the presence of IFNγ, NK cells migrated to the LN where they interacted with DCs promoting IL-27 production, leading to the suppression of disease-inducing T_H_17 T cells and therefore to protection from autoimmunity (137). Studies in MS patients revealed that the reduced cytolytic functions of NK cells are due to an impaired interaction between NK cells and CD4 T cells *via* DNAM-1-PVR and 2B4-CD48. Thus, the dysregulation of NK–T cell interaction in MS may present an attractive therapeutic target. Indeed, therapeutic immune modulation targeting IL-2Rα (CD25) by daclizumab was shown to selectively expand CD56^bright^ NK cells, activate NK cell functions, and induced upregulation of PVR on the surface of CD4 T cells, rendering these susceptible for NK cell-mediated killing (138–140).

The activating receptor NKp46 is known to be associated with the induction of type I diabetes. Using knockout mice, in which NKp46 is ablated, revealed that these mice are less prone to develop diabetes. Mechanistically, NK cells degranulated after binding *via* NKp46 to beta islet cells of the pancreas. The ligand for NKp46 expressed on beta islet cells is still unknown (141, 142). Whether the lack of NKp46 has also influence on autoreactive T cells in diabetes type I remains to be shown.

Moreover, NKp46 is also involved in graft-versus-host disease (GVHD) because the absence of NKp46 in GVHD led to rapid mortality. However, it has remained elusive if NKp46 is needed for direct killing of host-reactive T cells, or whether regulation occurs indirectly *via* APCs in the context of GVHD (143). Furthermore, NK cells expressing the activating receptor Ly49D rapidly killed allogenic DCs in a murine skin transplantation model through the release of perforin. The absence of allogenic DCs inhibited alloreactive CD8 T cell responses emerging in the draining LN. In addition, NK cells could limit alloreactive CD4 T cells *via* the activating receptor Ly49D (144). These studies demonstrate that NK cells play a role in GVHD *via* the activating receptors Ly49D and NKp46.

## Concluding Remarks

Even though NK cells are mainly known as killer cells of the innate immune system, there is more and more evidence that NK cells can shape the adaptive immune system by influencing T cells in different stages of their lifespan. During T cell priming, NK cells indirectly alter T cell responses by affecting DCs. Reciprocally, DCs are also able to modulate NK cells and this bidirectional interaction affects the emerging T cell response. More detailed insight into the detailed mechanisms of DC/NK interaction will be important to tailor T cell immunity in the context of vaccination or toleration.

The absence of NK cells induces alterations in the early phase of T cell responses, including direct attack of T cells. The detailed mechanisms of this direct regulation are, however, still being defined. Since NK cells are regulated *via* the net balance of signals perceived by their activating and inhibiting receptors, more insights into the regulation of NK receptor ligand expression on activated T cells is required.

Since T cells have evolved several mechanisms to shield themselves against NK-mediated killing during early activation, a more detailed molecular understanding about this shielding process is important. Such knowledge might also be useful to understand whether and how autoreactive T cells can be rendered targets for NK cell attack.

## Author Contributions

All authors listed have made substantial, direct, and intellectual contribution to the work and approved it for publication.

## Conflict of Interest Statement

The authors declare that the research was conducted in the absence of any commercial or financial relationships that could be construed as a potential conflict of interest.
